# *OsNAR2.1* Positively Regulates Drought Tolerance and Grain Yield Under Drought Stress Conditions in Rice

**DOI:** 10.3389/fpls.2019.00197

**Published:** 2019-02-21

**Authors:** Jingguang Chen, Tiantian Qi, Zhi Hu, Xiaoru Fan, Longlong Zhu, Muhammad Faseeh Iqbal, Xiaoming Yin, Guohua Xu, Xiaorong Fan

**Affiliations:** ^1^State Key Laboratory of Crop Genetics and Germplasm Enhancement, MOA Key Laboratory of Plant Nutrition and Fertilization in Low-Middle Reaches of the Yangtze River, Nanjing Agricultural University, Nanjing, China; ^2^CAAS-IRRI Joint Laboratory for Genomics-Assisted Germplasm Enhancement, Agricultural Genomics Institute in Shenzhen, Chinese Academy of Agricultural Sciences, Shenzhen, China

**Keywords:** drought tolerance, grain yield, water use efficiency, *OsNAR2.1*, rice

## Abstract

Drought is an important environmental factor that severely restricts crop production. The high-affinity nitrate transporter partner protein *OsNAR2.1* plays an essential role in nitrate absorption and translocation in rice. Our results suggest that *OsNAR2.1* expression is markedly induced by water deficit. After drought stress conditions and irrigation, compared with wild-type (WT), the survival rate was significantly improved in *OsNAR2.1* over-expression lines and decreased in *OsNAR2.1* RNAi lines. The survival rate of Wuyunjing7 (WYJ), *OsNRT2.1* over-expression lines and *OsNRT2.3a* over-expression lines was not significantly different. Compared with WT, overexpression of *OsNAR2.1* could significantly increase nitrogen uptake in rice, and *OsNAR2.1* RNAi could significantly reduce nitrogen uptake. Under drought conditions, the expression of *OsNAC10, OsSNAC1, OsDREB2a*, and *OsAP37* was significantly reduced in *OsNAR2.1* RNAi lines and increased substantially in *OsNAR2.1* over-expression lines. Also, the chlorophyll content, relative water content, photosynthetic rate and water use efficiency were decreased considerably in *OsNAR2.1* RNAi lines and increased significantly in *OsNAR2.1* over-expression lines under drought conditions. Finally, compared to WT, grain yield increased by about 9.1 and 26.6%, in *OsNAR2.1* over-expression lines under full and limited irrigation conditions, respectively. These results indicate that *OsNAR2.1* regulates the response to drought stress in rice and increases drought tolerance.

## Introduction

Abiotic stresses, particularly drought and nitrogen (N) deficiency, are important limiting factors for plant growth, development and agricultural production (Ding et al., 2018). Drought stress can seriously impair the growth and development of many soil plants and is often responsible for massive reductions in crop yields globally (Ahuja et al., 2010; Shekoofa and Sinclair, 2018). Drought stress decreases photosynthetic rate, restricts plant growth, and leads to a decrease in yield ranging from 13 to 94% (Farooq et al., 2009). Some studies have shown that the main reason for rice yield reduction caused by drought stress is the decrease in seed setting rate, the ratio of filled grains to total spikelets, and grain number per panicle (Ekanayake et al., 1989, 1990). Abiotic stresses are inevitable because plants need to grow in soil (Farooq et al., 2009). Therefore, improving the adaptability of plants to abiotic stress is of great significance for improving agricultural productivity (Ahuja et al., 2010; Chen G. et al., 2017).

Nitrogen (N) is an essential nutrient for plants; it affects all processes of rice from metabolism to growth and development (Xu et al., 2012). The absorption and utilization of water and nitrogen nutrition are two coupled physiological processes (Shangguan et al., 2000; Ding et al., 2018). Supplying plants with N can increase drought resistance by increasing root hydraulic conductivity (Lpr) through increased ABA and aquaporin expression (Chaves et al., 2002; Guo et al., 2007; Shatil-Cohen et al., 2011; Ren et al., 2015; Ding et al., 2016). Taken together, these data suggest that increasing N uptake may be beneficial during drought conditions.

*OsNAR2.1* is a high affinity nitrate transporter partner protein which, along with *OsNRT2s*, plays a central role in nitrate absorption and translocation in rice (Yan et al., 2011; Liu et al., 2014). Chen et al. (2016) showed that increased expression of *OsNRT2.1*, using the *OsNAR2.1* promoter, could improve yield and ANUE in rice. Tang et al. (2012) reported the involvement of rice *OsNRT2.3a* in the transport of NO_3_^−^ from root to shoot. Yan et al. (2011) reported that knockdown of *OsNAR2.1* by RNA interference (RNAi) could suppress *OsNRT2.1* and *OsNRT2.3a* expression in mutant roots and demonstrated that *OsNAR2.1* performs a crucial function in both high and low nitrate absorption. Chen J. et al. (2017) showed that using the *OsNAR2.1* promoter to drive *OsNAR2.1* expression could improve nitrate and ammonium absorption and yield in rice. However, the mechanisms by which *OsNAR2.1* and *OsNRT2s* modify drought resistance are not well known. To date, some studies have suggested that the overexpression of stress-related genes may improve drought tolerance in rice to some extent (Kim et al., 2009; Lu et al., 2013). Despite a few such efforts to develop drought-tolerant rice plants, very few have shown an improvement in grain yields under field conditions. In this study, the contributions of *OsNAR2.1* and *OsNRT2s* to drought stress regulation were studied, including effects on crop yield, stress-related genes, and photosynthesis rate.

## Materials and Methods

### Plant Materials

The generation and basic molecular properties of *OsNRT2.1/OsNRT2.2* over-expression transgenic lines (OE1, OE2, OE3) was previously described in Chen et al. (2016). The genetic background of *OsNRT2.1* over-expression transgenic lines was cv. Wuyunjing7 (WYJ). The generation and basic molecular properties of *OsNAR2.1* RNAi transgenic lines (r1, r2) was previously described in Yan et al. (2011). The genetic background of *OsNAR2.1* RNAi transgenic lines was cv. Nipponbare (WT).

We amplified the *OsNAR2.1* or *OsNRT2.3a* open reading frame sequence from cDNA isolated from *Oryza sativa* L. ssp. Japonica cv. Nipponbare using the primers listed in Supplementary Table S1. The PCR products were cloned into the pMD19-T vector (TaKaRa) and confirmed by restriction enzyme digestion and DNA sequencing. The restriction sites (*Asc*I and *Kpn*I) or (*Spe*I and *Spe*I) were incorporated into primers to facilitate cloning into the *pUbi:OsNAR2.1* vector or the *p35S:OsNRT2.3a* vector, respectively. These constructs were introduced into *Agrobacterium tumefaciens* strain EHA105 by electroporation and then transformed into cv. Nipponbare or cv. Wuyunjing7 as described previously (Chen et al., 2016) (Supplementary Figure S1).

### Growth Conditions

To determine seedling survival under drought stress, the seedlings were grown for 21 days under highly irrigated conditions by maintaining 10 equal-sized seedlings per pot. Irrigation was withheld for 12 days, followed by re-watering for 7 days. Photographs were taken before the stress (0 days), at the end of the stress period, and a week after recovery. Five biological replicates were maintained among all lines.

For hydroponic drought stress experiments, rice seedlings were grown with the normal IRRI solution for 2 weeks and then transferred to nutrient solution supplemented with 10% (w/v) PEG6000 for 2 weeks. The hydroponic experiments were carried out in a growth room with a 14 h light (30°C) (8:00 – 22:00)/10 h dark (22°C) (22:00 – 8:00) photoperiod and 60% relative humidity. In all treatments, nutrient solutions were replaced every 2 days. At the end of the drought stress experiments, measurements of chlorophyll content, relative water content, photosynthesis rate and water use efficiency were recorded.

The rice plants for the limited water supply experiment were cultivated in plots at the experimental site of Nanjing Agricultural University, Nanjing, Jiangsu, China. The plots size was 1 m × 1 m and the seedlings were planted in a 5 × 5 array. Urea was used as nitrogen fertilizer, and 180 kg N/ha of fertilizer was applied on each plot. All plants were normally irrigated for 2 weeks after transplanting. Then limited water supply (60% field capacity) was applied to the experimental group and fully irrigated conditions (100% field capacity) was applied to controls until completion of the life cycle, and its agronomic characteristics were analyzed.

### RNA Extraction and qRT-PCR Assay

RNA was extracted from the root or shoot of seedlings under well-watered and drought stress conditions. Total RNA was extracted using TRIzol reagent (Vazyme Biotech Co., Ltd., China). DNase I-treated total RNAs were subjected to reverse transcription (RT) with HiScript II Q Select RT SuperMix for qPCR (+gDNA wiper) kit (Vazyme Biotech Co.). Triplicate quantitative assays were performed using the 2 × T5 Fast qPCR Mix (SYBRGreenI) kit (TsingKe Co, Ltd., China). The primers for qRT-PCR are shown in Supplementary Table S2.

### DNA Extraction and Southern Blot Analysis

Genomic DNA was extracted from leaves of WT and transgenic plants using the SDS method (Chen et al., 2016). 60 μg of DNA was digested with *EcoR*I and *Hind*III restriction enzymes 10 h at 37°C. The digested DNA was separated on a 1% (w/v) agarose gel and transferred to a Hybond-N^+^ nylon membrane, and hybridized with the coding sequence of hygromycin-resistance gene as hybridization probe (Chen et al., 2016).

### Stress Response Measurements

The degree of relative chlorophyll content in the fully expanded last leaf was determined using a SPAD-502 Chlorophyll Meter (Minolta Co.). Relative water content was measured as described (Ramegowda et al., 2014) in the leaves for chlorophyll content measurements. Relative water content = (fresh weight - dry weight)/(turgid weight - dry weight) × 100%.

The same leaf is used to measure photosynthetic rate. Photosynthesis rates were measured in rice seedlings using a Li-COR6400 portable photosynthesis system equipped with a LED leaf cuvette (Li-COR, Lincoln, NE, United States). Water use efficiency was calculated using photosynthesis measurements and the transpiration rate (Ramegowda et al., 2014). Water use efficiency = photosynthesis/transpiration rate.

### Determination of Root ^15^N-NO_3_^−^ and ^15^N-NH_4_^+^ Influx Rate

Rice seedlings of WT and transgenic plants were grown in IRRI nutrient solution containing 1 mM NH_4_^+^ for 2 weeks and then nitrogen starved for 5 days. The plants were transferred first to 0.1 mM CaSO_4_ for 1 min, then to a complete nutrient solution containing 1.25 mM ^15^NH_4_NO_3_ or 1.25 mM NH_4_^15^NO_3_ (atom % ^15^N: ^15^NO_3−_, 99%; ^15^NH_4_^+^, 99%) for 5 min, and finally to 0.1 mM CaSO_4_ for 1 min. And the ^15^N influx rate was calculated following the method in Chen J. et al. (2017).

### Statistical Analysis

All data were analyzed by ANOVA using the statistical SPSS 10 program (SPSS Inc., Chicago, IL, United States). The different letters indicate a significant difference between the transgenic line and the WT (*P* < 0.05, one-way ANOVA).

## Results

### The Response of *OsNAR2.1* and *OsNRT2s* Relative Expression to Drought Stress

**FIGURE 1:**
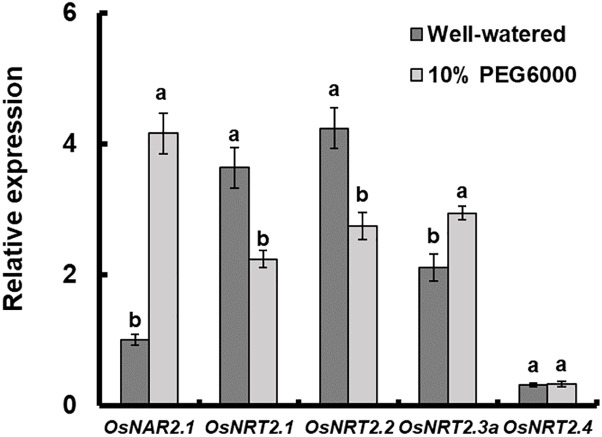
Relative expression analysis of *OsNAR2.1* and *OsNRT2s* under drought stress treatment by real-time RT-PCR. Rice seedlings were supplied with normal IRRI solution for 7 days and then transferred to nutrient solution containing 10% (w/v) PEG6000 for 3 days. RNA was extracted from roots of rice cv. Nipponbare. Error bars: SE (*n* = 3 plants).

The expression of *OsNAR2.1* and *OsNRT2s* under drought stress was analyzed by qRT-PCR. The results showed that the expression of *OsNAR2.1*, *OsNRT2.1*, *OsNRT2.2*, and *OsNRT2.3a* was affected by 10% PEG6000 treatment and *OsNRT2.4* did not show any response to drought stress (Figure 1). Expression of *OsNAR2.1* and *OsNRT2.3a* was increased by 10% PEG6000. As a result, the expression of *OsNRT2.1* and *OsNRT2.2* was inhibited. Among them, *OsNAR2.1* expression showed the most drastic change under drought stress, the expression increased by nearly 4 times that of fully irrigated conditions (Figure 1).

**FIGURE 2:**
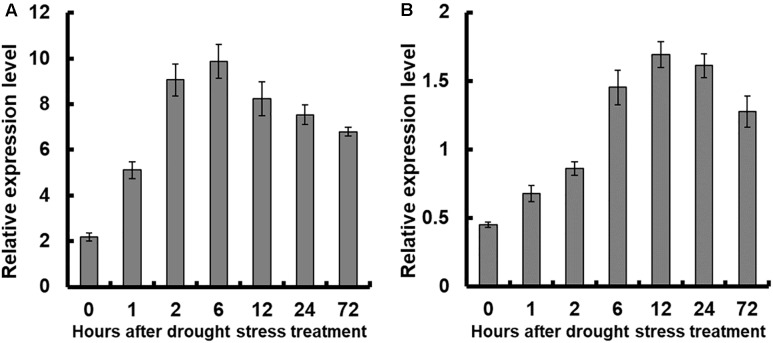
Relative expression analysis of *OsNAR2.1* under drought stress treatment by real-time RT-PCR. Rice seedlings were supplied with normal IRRI solution for 7 days then transferred to nutrient solution containing 10% (w/v) PEG6000 for different time. RNA was extracted from **(A)** roots and **(B)** shoots of rice cv. Nipponbare. Error bars: SE (*n* = 3 plants).

Results showed that the expression of *OsNAR2.1* was significantly enhanced in both shoots and roots of plants treated with 10% PEG6000 (Figure 2). Overall, *OsNAR2.1* expression in roots was significantly higher than in shoots, which was consistent with published work by Feng et al. (2011). In roots, *OsNAR2.1* expression increased after 10% PEG6000 application and reached maximal expression levels after 6 h, followed by a gradual decline with prolonged treatment (12–72 h) to a 3 to 5-fold increase (Figure 2A). In shoots, the expression of *OsNAR2.1* reached the highest level after 12 h of treatment, with a 3.5-fold increase (Figure 2B).

### Identification of *OsNAR2.1* and *OsNRT2s* Transgenic Lines

The *OsNRT2.1* over-expression transgenic lines were designated as OE1, OE2 and OE3 and *OsNAR2.1* RNAi transgenic lines as r1 and r2 previously used in Chen et al. (2016) and Yan et al. (2011). We also performed molecular verification to confirm these lines. qRT-PCR analysis showed that the expression of *OsNRT2.1* in OE1, OE2 and OE3 increased significantly as compared with WYJ, (Supplementary Figure S2A). Southern blot analysis showed that *OsNAR2.1* RNAi transgenic lines r1 and r2 are two independent lines (Supplementary Figure S3A) and, compared with WT, the expression of *OsNAR2.1* in root and shoot of r1 and r2 decreased significantly (Supplementary Figure S3C).

We used the *Agrobacterium tumefaciens-mediated* method to generate transgenic lines. The *p35S:OsNRT2.3a* expression vector was inserted into Wuyunjing7 (*O. sativa* L. ssp. Japonica cv., WYJ) and the *pUbi:OsNAR2.1* expression vector was inserted into Nipponbare (*O. sativa* L. ssp. Japonica cv., WT). On the basis of Southern blot analysis and RNA expression level, we selected three independent lines of *p35S:OsNRT2.3a* designated as WA1, WA2, and WA3 (Supplementary Figures S2B,C), two independent lines of *pUbi:OsNAR2.1* designated as Ox1 and Ox2 (Supplementary Figures S3B,D).

### Drought Stress Sensitivity of *OsNAR2.1* and *OsNRT2s* Transgenic Lines at the Seedling Stage

**FIGURE 3:**
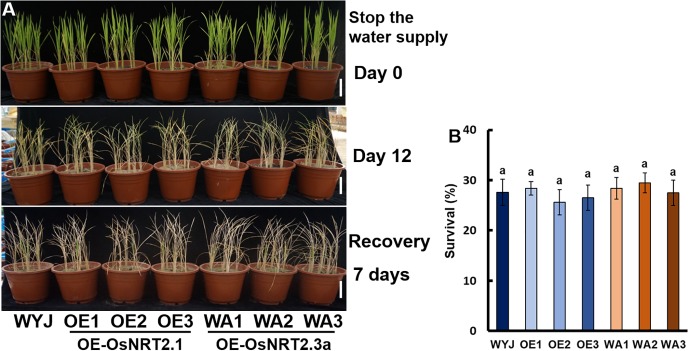
Drought stress sensitivity of *OsNRT2s* transgenic lines at the seedling stage. The seedlings were grown for 21 days in full irrigation by maintaining 10 equal-sized seedlings per pot. Irrigation was withheld for 14 days, followed by re-irrigation for 7 days. **(A)** Phenotype of drought-stressed plants followed by recovery. **(B)** Seedling survival after recovery, the number of seedlings with at least one fully expanded leaf was counted. Error bars: SE (*n* = 5 pots). The different letters indicate a significant difference between the transgenic line and WT (*P* < 0.05, one-way ANOVA).

To test the involvement of *OsNRT2.1, OsNAR2.1*, and *OsNRT2.3a* in drought tolerance, the drought stress response of their transgenic lines was tested at the vegetative stage. Irrigation was withheld for 12 days, followed by re-watering for 7 days (Figure 3A). The survival rate of WYJ, *OsNRT2.1* and *OsNRT2.3a* over-expression transgenic lines was not significantly different and was about 27% (Figure 3B).

**FIGURE 4:**
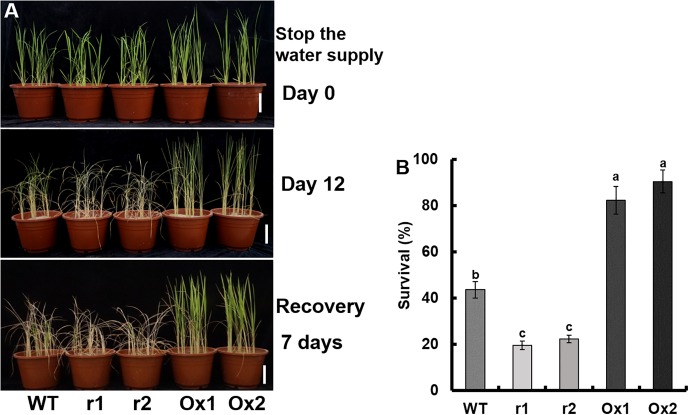
Drought stress sensitivity of *OsNAR2.1* transgenic lines at the seedling stage. The seedlings were grown for 21 days in full irrigation by maintaining 10 equal-sized seedlings per pot. Irrigation was withheld for 12 days, followed by re-irrigation for 7 days. **(A)** Phenotype of drought-stressed plants followed by recovery. **(B)** Seedling survival after recovery, the number of seedlings with at least one fully expanded leaf was counted. Error bars: SE (*n* = 5 pots). The different letters indicate a significant difference between the transgenic line and WT (*P* < 0.05, one-way ANOVA).

However, in *OsNAR2.1* transgenic lines (Figure 4A), the change in survival rate was significant. The recovery rate in WT was approximately 43.6%, in *OsNAR2.1* RNAi transgenic lines was 21.0%, and in *OsNAR2.1* over-expression transgenic lines was 86.3% (Figure 4B).

### Effect of *OsNAR2.1* Expression on Rice Growth at Seedling Stage Under Drought Stress

To further assess the effect of *OsNAR2.1* on growth response to drought stress, the seedlings of *OsNAR2.1* transgenic lines were grown in normal IRRI solution for 14 days and then transferred into the nutrient solution containing 10% PEG6000 for 14 days, nutrient solution without PEG6000 was used as a control (Supplementary Figures S4A,B). Under drought conditions, wilting and foliar chlorosis was observed in WT and *OsNAR2.1* RNAi lines but not in *OsNAR2.1* over-expression lines (Supplementary Figure S4C).

**FIGURE 5:**
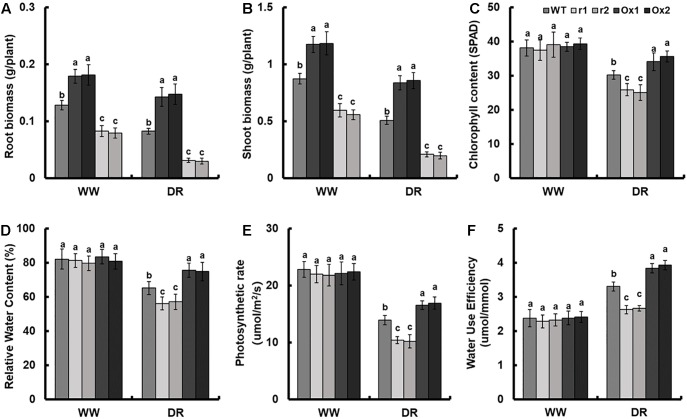
Growth parameters of *OsNAR2.1* transgenic lines under well-watered and drought stress conditions. Rice seedlings were grown in normal IRRI solution for 14 days and then transferred to nutrient solution containing 10% (w/v) PEG6000 for 14 days. **(A)** Root biomass, **(B)** shoot biomass, **(C)** chlorophyll content, **(D)** relative water content, **(E)** photosynthesis rate and **(F)** water use efficiency were assayed. Relative Water Content = (fresh weight – dry weight)/(turgid weight – dry weight) × 100%. Water Use Efficiency (umol/mmol) = photosynthesis/transpiration rate. WW, well-watered; DR, drought stress (10% PEG6000). Error bars: SE (*n* = 5 plants). The different letters indicate a significant difference between the transgenic lines and WT (*P* < 0.05, one-way ANOVA).

Compared to control, drought stress led to the suppression of root and shoot growth in all plants (Figure 5A,B and Supplementary Figure S4D). As compared with WT, the biomass per plant of *OsNAR2.1* RNAi lines was reduced by 34.2% and that of *OsNAR2.1* over-expression lines was increased by 35.7% in normal solution (Supplementary Figure S4D). However, under drought stress, the biomass of *OsNAR2.1* RNAi lines was reduced by 60.3% and in *OsNAR2.1* over-expression lines biomass was increased by 68.2% as compared with WT (Supplementary Figure S4D). Compared with WT, the roots biomass of *OsNAR2.1* RNAi lines was reduced by 40.7% in normal solution and by 76.1% under drought stress (Figure 5A). The shoots biomass of *OsNAR2.1* RNAi lines was reduced by 35.1% in normal solution and by 66.9% under drought stress (Figure 5B). Similarly, compared with WT, the roots biomass of *OsNAR2.1* over-expression lines was increased by 36.8% in normal solution and by 62.8% under drought stress (Figure 5A). Moreover, the shoots biomass of *OsNAR2.1* over-expression lines increased by 33.8% in normal solution and by 59.9% under drought stress (Figure 5B).

There was no significant difference in chlorophyll content, relative water content, photosynthesis rate or water use efficiency between *OsNAR2.1* RNAi lines and *OsNAR2.1* over-expression lines under normal solution (Figure 5C–F). However, after 14 days of drought stress as compared with WT, the chlorophyll content of *OsNAR2.1* RNAi lines was reduced by 15.6% and the *OsNAR2.1* over-expression lines was increased by 14.5% (Figure 5C). Under drought stress, the *OsNAR2.1* RNAi lines showed a reduction in relative water content of about 13.1% compared with WT, but *OsNAR2.1* over-expression lines showed an increase of 15.5% (Figure 5D). Compared with WT, the photosynthesis rate of *OsNAR2.1* RNAi lines was reduced by 26.1% and the *OsNAR2.1* over-expression lines was improved by 20.1% following drought stress (Figure 5E). Water use efficiency of WT in normal conditions was significantly lower than that under drought conditions (Figure 5F). The *OsNAR2.1* RNAi lines showed a reduction in water use efficiency by 19.9% as compared with drought-stressed WT and *OsNAR2.1* over-expression lines showed an increase of 17.5% (Figure 5F).

### *OsNAR2.1* Transgenic Lines Altered Expression of Stress-Responsive Genes Under Drought Conditions

**FIGURE 6:**
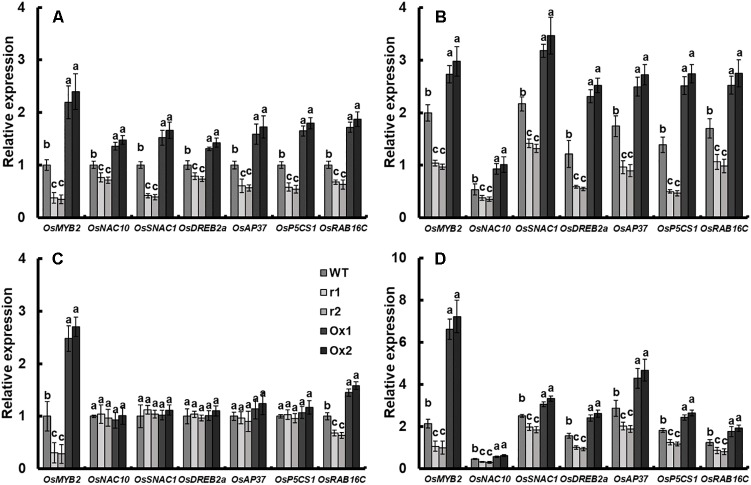
Expression of stress-responsive genes in *OsNAR2.1* transgenic lines. Growth conditions and treatments were the same as described in Figure 4. RNA was extracted from root of seedlings under **(A)** well-watered and **(B)** drought stress (10% PEG6000) conditions. RNA was extracted from shoot of seedlings under **(C)** well-watered and **(D)** drought stress (10% PEG6000) conditions. Error bars: SE (*n* = 3 plants). The different letters indicate a significant difference between the transgenic line and WT (*P* < 0.05, one-way ANOVA).

To understand the role of *OsNAR2.1* in drought stress responses, we analyzed the expression of several known genes involved in abiotic and biotic stress in *OsNAR2.1* transgenic lines, including *OsMYB2* (Yang et al., 2012), *OsNAC10* (Jeong et al., 2010), *OsSNAC1* (Hu et al., 2006), *OsDREB2a* (Cui et al., 2011; Mallikarjuna et al., 2011), *OsAP37* (Oh et al., 2009; Ramegowda et al., 2014), *OsP5CS1* (Sripinyowanich et al., 2013) and *OsRAB16C* (Chen et al., 2015). Under normal conditions as compared with WT, the expression of all of these genes in the roots of *OsNAR2.1* RNAi lines was decreased and in *OsNAR2.1* over-expression lines was increased (Figure 6A). Moreover, in shoots, only *OsMYB2* and *OsRAB16C* expression responded to *OsNAR2.1* manipulation, showing a decrease in *OsNAR2.1* RNAi lines and an increase in *OsNAR2.1* over-expression lines. Conversely, no significant difference was found in the expression of non-stress related genes in transgenic lines (Figure 6C). Under drought stress, the expression of all of these genes was significantly decreased in the roots or shoots of *OsNAR2.1* RNAi lines and significantly increased in *OsNAR2.1* over-expression lines (Figure 6B,D).

### Effect of *OsNAR2.1* Expression on Rice Grain Yield Under Drought Stress

**FIGURE 7:**
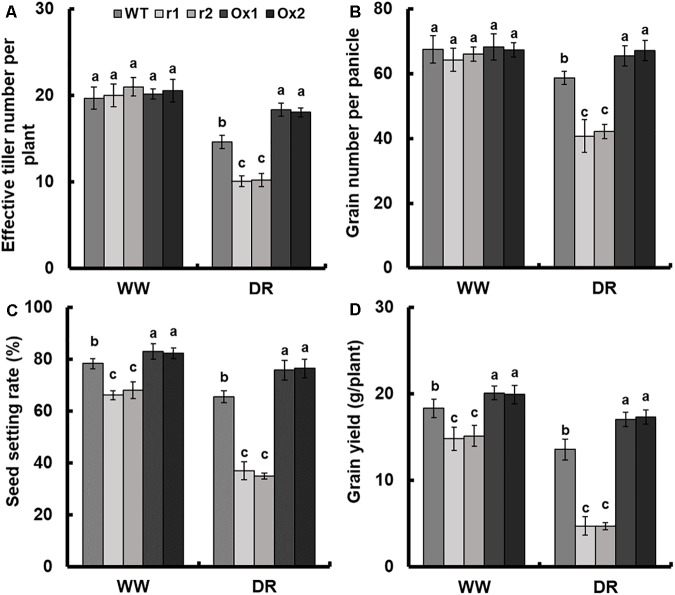
Comparison of grain yield between *OsNAR2.1* RNAi lines and *OsNAR2.1* over-expression lines in limited and fully irrigated conditions. All plants were normally irrigated for 2 weeks after transplantation. Then limited water supply (60% field capacity) was applied to experimental groups and maintained throughout the experiment until completion of the life cycle, full irrigation (100% field capacity) was applied to controls. Then agronomic traits were assayed. WW: (full irrigation); DR: drought stress (limited water supply). **(A)** Effective tiller number per plant, **(B)** grain number per panicle, **(C)** seed setting rate and **(D)** grain yield. Error bars: SE (*n* = 5 plants). The different letters indicate a significant difference between the transgenic line and WT (*P* < 0.05, one-way ANOVA).

Detailed analysis of drought response in *OsNAR2.1* transgenic lines was performed by maintaining plants at 60% field capacity (Supplementary Figure S5), the yield of transgenic lines was measured by comparing the results with the fully irrigated field which we designated as a normal field. The seed setting rate and the grain number per panicle of WT under limited irrigation were 16.5 and 13.1%, respectively, lower than those of fully irrigated field. As a result, grain weight was reduced by 23.3% and grain yield was reduced by 26% (Figure 7B–D and Supplementary Figure S6D). Moreover, the yield of *OsNAR2.1* over-expression lines under limited irrigation was not significantly different from the WT under full irrigation (Figure 7D).

As compared with WT, the effective tiller number and grain number showed no difference in transgenic lines in the normal field. However, in the drought field, *OsNAR2.1* RNAi lines showed a decrease of 30.6 and 29.8%, respectively and *OsNAR2.1* over-expression lines increased by 24.4 and 13%, respectively (Figure 7A,B). Compared with WT, the seed setting rate and grain yield of RNAi lines was decreased by 14.3 and 18.2%, respectively in the normal field. In addition, as compared with WT, the seed setting rate and grain yield were reduced by 45 and 65.2% in RNAi lines under drought stress. However, the seed setting rate and grain yield in *OsNAR2.1* over-expression lines were increased by 5.6 and 9.1% in the normal field as compared to WT. Under drought conditions the *OsNAR2.1* over-expression lines, seed setting rate and grain yield was increased by 16.3 and 26.6%, respectively (Figure 7C and Supplementary Figure S6D).

We also analyzed other agronomic traits amongst *OsNAR2.1* transgenic lines. When there was plentiful irrigation available, the total tiller number per plant and 1000-grains weight of transgenic lines of *OsNAR2.1* showed no difference. However, in limited water supply a 13.2% decline and 11.5% increase were observed in *OsNAR2.1* RNAi lines and *OsNAR2.1* over-expression lines, respectively, for tiller number and a similar pattern was observed in 1000-grain weight as compared to WT (Supplementary Figures S6A,C). When full irrigation was applied, panicle length declined in *OsNAR2.1* RNAi lines and increased in *OsNAR2.1* over-expression lines by about 19.0 and 25.8%, respectively, for grain weight it was 15.8 and 8.6%, respectively, for dry weight it was 11.1 and 8.4%, respectively as compared to WT. In contrast, when water supply was limited panicle length declined in *OsNAR2.1* RNAi lines and increased in *OsNAR2.1* over-expression lines by 39.1 and 25.8%, respectively, for grain weight it was 50.4% decrease and 31.5% increase, respectively, for dry weight it was 50.0 and 17.1%, respectively. Furthermore, the grain/straw ratio in *OsNAR2.1* RNAi lines decreased by 43.5% and in *OsNAR2.1* over-expression lines increased by 17.4% under drought conditions (Supplementary Figure S6).

### Rates of NO_3_^−^ and NH_4_^+^ Influx in WT and Transgenic Plants

We analyzed short-term NO_3_^−^ and NH_4_^+^ uptake in same-size seedlings of the transgenic lines by exposing the plants to 1.25 mM NH_4_^15^NO_3_ or 1.25 mM ^15^NH_4_NO_3_ for 5 min to determine the effect of *OsNRT2.1*, *OsNRT2.3a* or *OsNAR2.1* expression on root NO_3_^−^ and NH_4_^+^ influx into intact plants. Compared with WYJ, overexpression of *OsNRT2.1* increased NH_4_^15^NO_3_ and ^15^NH_4_NO_3_ influx by 29.4% (Supplementary Figure S7A) and 12.6% (Supplementary Figure S7B), the ratio of ^15^NH_4_^+^ to ^15^NO_3_^−^ influx decreased by 12.9% (Supplementary Figure S7C). Compared with WYJ, overexpression of *OsNRT2.3a* did not significantly affect the influx of NH_4_^15^NO_3_ and ^15^NH_4_NO_3_ by rice roots at seedling stage (Supplementary Figures S7A,B). As compared with WT, the NH_4_^15^NO_3_ and ^15^NH_4_NO_3_ influx of *OsNAR2.1* RNAi lines decreased by 43.0% and 46.0%, that of *OsNAR2.1* over-expression lines increased by 32.8% and 30%, there was no significant difference in the ratio of ^15^NH_4_^+^ to ^15^NO_3_^−^ influx (Supplementary Figures S7D–F).

We also analyzed *OsNAR2.1* and *OsNRT2s* expression in *OsNRT2.1*, *OsNRT2.3a* or *OsNAR2.1* transgenic lines. Compared with WYJ, the expression of *OsNAR2.1* increased significantly in *OsNRT2.1* over-expression lines, but there was no significant difference in the expression of *OsNRT2.2*, *OsNRT2.3a* and *OsNRT2.4* (Supplementary Figure S8A). There was no significant difference in the expression of *OsNAR2.1, OsNRT2.2* and *OsNRT2.4* between *OsNRT2.3a* over-expression lines and WYJ (Supplementary Figure S8B). The expression of *OsNRT2.1*, *OsNRT2.2* and *OsNRT2.3a* decreased in *OsNAR2.1* RNAi lines, increased in *OsNAR2.1* over-expression lines, and the expression of *OsNRT2.4* is not affected by *OsNAR2.1* (Supplementary Figure S8C).

## Discussion

Rice (*Oryza sativa* L.) is the staple food for more than half of the world’s population. Therefore, an increase rice production will be needed to meet the growing demand of future populations (Sharp et al., 2004). Previous studies have shown that drought stress strongly affects plant growth and nitrogen metabolism (Allahverdiyev, 2016). Under drought stress, adjusting nitrogen supply can enhance the adaptability of crops by improving water relationship (Chaves et al., 2002; Guo et al., 2007).

*OsNAR2.1* is a high affinity nitrate transporter partner protein. Yan et al. (2011) reported that the *OsNAR2.1* plays a key role in both high and low nitrate absorption. (Chen J. et al. (2017)) found that *pOsNAR2.1:OsNAR2.1* expression enhances nitrogen uptake efficiency and grain yield in transgenic rice plants. In this study, we examined the possibility of *OsNAR2.1* participating in regulation of the response to drought stress and its effect on rice yield by using *OsNAR2.1* RNAi and *OsNAR2.1* over-expression transgenic lines.

### *OsNAR2.1* Involvement in Drought Tolerance of Rice Seedlings

Many studies have shown that nitrogen supply has an essential influence on the response of plants to drought stress (Chaves et al., 2002; Guo et al., 2007; Ding et al., 2018). *OsNRT2.1* is a high affinity nitrate transporter, mainly expressed at the root and responsible for nitrate uptake (Katayama et al., 2009; Feng et al., 2011). Previous studies have shown that overexpression of *OsNRT2.1* increases the expression of *OsNAR2.1* and this may be responsible for increased nitrogen uptake (Chen et al., 2016; Luo et al., 2018). This is consistent with our data showing that compared with WYJ, overexpression of *OsNRT2.1* increased NH_4_^15^NO_3_ influx by 29.4% (Supplementary Figure S7A) and the expression of *OsNAR2.1* increased significantly in *OsNRT2.1* over-expression lines (Supplementary Figure S8A). Increased nitrate uptake could promote ammonium uptake by rice (Duan et al., 2006; Li et al., 2006). However, although ^15^NH_4_NO_3_ influx of *OsNRT2.1* overexpression lines increased by 12.6% as compared with WYJ (Supplementary Figure S7C), the ratio of ^15^NH_4_^+^ to ^15^NO_3_^−^ influx decreased by 12.9% (Supplementary Figure S7C).

Tang et al. (2012) reported that *OsNRT2.3a* is responsible for the transport of nitrate from root to shoot. Previous studies have shown that overexpression of *OsNRT2.3a* did not increase ^15^NH_4_^15^NO_3_ uptake in rice (Fan et al., 2016). This is consistent with our data showing compared with WYJ, overexpression of *OsNRT2.3a* did not significantly affect the influx of NH_4_^15^NO_3_ and ^15^NH_4_NO_3_ by rice roots at seedling stage (Supplementary Figures S7A,B). This is likely due to the fact that overexpression of *OsNRT2.3a* did not lead to a significant increase in expression of *OsNAR2.1* (Supplementary Figure S8B). Previous work has shown that overexpression of *OsNAR2.1* increase nitrate and ammonium absorption in rice, and increase rice biomass under 1.25 mM NH_4_NO_3_ treatment (Chen J. et al., 2017). These results are consistent with our data showing as compared with WT, the NH_4_^15^NO_3_ and ^15^NH_4_NO_3_ influx of *OsNAR2.1* RNAi lines was reduced by 43.0 and 46.0%, that of *OsNAR2.1* over-expression lines was increased by 32.8 and 30%, there was no significant difference in the ratio of ^15^NH_4_^+^ to ^15^NO_3_^−^ influx (Supplementary Figures S7D–F). Also, the expression of *OsNRT2.1*, *OsNRT2.2* and *OsNRT2.3a* decreased in *OsNAR2.1* RNAi lines and increased in *OsNAR2.1* over-expression lines (Supplementary Figure S8C).

Photosynthesis is the main source of biomass accumulation in all plants, it is also one of the most sensitive physiological processes during abiotic stress (DaMatta et al., 2002). Drought stress can inhibit stomatal opening and photosynthesis, and can lead to an increase in water use efficiency (Silva et al., 2010; Waraich et al., 2011; Allahverdiyev, 2016). Compared with WT, there were no significant differences in chlorophyll content, relative water content, photosynthesis rate and water use efficiency between *OsNAR2.1* RNAi lines and *OsNAR2.1* over-expression lines in well-watered conditions (Figure 5C–F). After 14 days of drought stress as compared with WT, the photosynthesis rate of *OsNAR2.1* RNAi lines decreased by 26.1%, and of *OsNAR2.1* over-expression lines increased by 20.1% (Figure 5E). *OsNAR2.1* RNAi lines showed a 19.9% reduction in water use efficiency compared with drought-stressed WT and *OsNAR2.1* over-expression lines increased efficiency by 17.5% (Figure 5E). Because of changes in nitrogen uptake, photosynthetic rate and water use efficiency, compared with WT, the biomass of *OsNAR2.1* RNAi decreased by 34.2% under normal solution and 60.3% under drought stress, the biomass of *OsNAR2.1* over-expression lines increased by 35.7% under normal solution and 68.2% under drought stress (Figure 5A,B and Supplementary Figure S4D).

There are two kinds of nitrogen applications for rice plants: ammonium and nitrate, with different nitrogen supply patterns and effects on plant growth. The root activity of rice with ammonium was significantly higher than that with nitrate, which might be related to the characteristics of aquaporins (Siemens and Zwiazek, 2003). Plants supplied with NH_4_^+^ had a higher assimilation rate and stomatal conductance than those applied with NO_3_^−^ (Guo et al., 2002). Compared with nitrate nutrition, ammonium nutrition increases photosynthesis rate under water stress at the early development stage of rice (Guo et al., 2007) and enhances the tolerance of rice seedlings to drought conditions (Li et al., 2009). Under drought stress, rice seedlings grow better in ammonium nutrition (Guo et al., 2002, 2007). Under PEG6000 treatment water stress, plants under NO_3_^−^ treatment had significantly lower chloroplast content than those under NH_4_^+^ treatment (Li et al., 2012; Xiong et al., 2017). Wilkinson et al. (2007) observed that the decrease in stomatal closure and leaf elongation rate was more sensitive to drought stress in maize plants supplied with high nitrate. Compared with nitrate supply, ammonium can significantly improve the drought resistance of rice. In the present study, irrigation was withheld for 12 days, followed by re-watering for 7 days (Figure 3A, 4A). The survival rate of WYJ, *OsNRT2.1* transgenic lines and *OsNRT2.3a* transgenic lines was not significantly different, about 27% (Figure 3B). Approximately 43.6% of the WT recovered, 21.0% of the *OsNAR2.1* RNAi transgenic lines recovered, and 86.3% of the *OsNAR2.1* over-expression transgenic lines recovered (Figure 4B). The results showed that over-expression of *OsNRT2.1* could increase ammonium uptake by rice, but at the same time increased more nitrate uptake (Supplementary Figure S7C), which might counteract the positive effect of ammonium on rice drought resistance. Overexpression of *OsNRT2.3a* did not increase nitrogen uptake or drought tolerance in rice. Compared with WT, the NH_4_^15^NO_3_ and ^15^NH_4_NO_3_ influx of *OsNAR2.1* RNAi lines decreased significantly, and that of *OsNAR2.1* over-expression lines increased significantly, there was no significant difference in the ratio of ^15^NH_4_^+^ to ^15^NO_3_^−^ influx (Supplementary Figures S7D–F). This ability to increase uptake of NH_4_^+^ as well as NO_3_^−^ may play a role in the mechanism of *OsNAR2.1* induced drought tolerance in rice.

We analyzed the expression of several known genes involved in abiotic and biotic stress in *OsNAR2.1* transgenic lines, including *OsMYB2, OsNAC1, OsSNAC1, OsDREB2a, OsAP37, OsP5CS1*, and *OsRAB16C*. Under normal conditions as compared with WT, the expression of all of these genes in the roots of *OsNAR2.1* RNAi lines was decreased and in *OsNAR2.1* over-expression lines was increased (Figure 6A). Conversely, no significant difference was found in the expression of other genes in transgenic lines (Figure 6C). In drought stress, the expression of all of these genes was significantly decreased in the roots or shoots of *OsNAR2.1* RNAi lines and increased in *OsNAR2.1* over-expression lines (Figure 6B,D). Previous studies have shown that overexpression of these genes increases drought tolerance in rice (Hu et al., 2006; Kim et al., 2009; Oh et al., 2009; Jeong et al., 2010; Cui et al., 2011; Mallikarjuna et al., 2011; Yang et al., 2012; Ramegowda et al., 2014; Chen et al., 2015). Overexpression of *OsNAR2.1* increased ammonium uptake in rice, while *OsNAR2.1* RNAi decreased ammonium uptake (Supplementary Figures S7E,F). Ammonium supply significantly increased abscisic acid (ABA) accumulation in plants (Zdunek and Lips, 2001; Rahayu et al., 2005; Fernández-Crespo et al., 2015; Ding et al., 2016). Under drought stress, ABA accumulation is essential to regulate the expression of drought-tolerant genes (Jang et al., 2004; Schraut et al., 2005; Lian et al., 2006; Mahdieh and Mostajeran, 2009; Parent et al., 2009), promote root water uptake and plant growth (Sharp, 2002; Zhang et al., 2006). *OsNAR2.1* regulates the expression of these drought regulating genes in rice under stress (Figure 6B,D); suggesting that the *OsNAR2.1* mediated increase in their expression is involved in rice drought tolerance.

### Effect of *OsNAR2.1* Expression on Rice Grain Yield Under Drought Stress

Compared with fully irrigated conditions, the grain yield of WT decreased by 26% under limited water supply (Figure 7D). The seed setting rate and grain number per panicle of WT in limited water supply were 16.5 and 13.1% lower than those in full irrigation; resulting in a 23.3% reduction in grain weight (Figure 7B,C and Supplementary Figure S6D). This agrees with previous studies showing that drought conditions impair pollen germination, seed setting rate, and grain number per panicle, resulting in decreased grain yield (Ekanayake et al., 1989, 1990; Saini and Westgate, 1999; Kim et al., 2009; Guan et al., 2010).

Drought stress also reduced the tiller number of rice (Kang et al., 2017). Compared with WT, the effective tiller number of *OsNAR2.1* RNAi lines and *OsNAR2.1* over-expression lines had no significant difference under complete watering conditions. The effective tiller number of *OsNAR2.1* RNAi lines decreased by 30.6% and *OsNAR2.1* over-expression lines increased by 24.4% under limited water supply (Figure 7A). Compared with WT, there was no significant difference in the grain number per panicle between *OsNAR2.1* RNAi lines and *OsNAR2.1* over-expression lines under complete watering conditions, but under limited water supply, the grain number per panicle of *OsNAR2.1* RNAi lines decreased by 29.8% and that of *OsNAR2.1* over-expression lines increased by 13.0% (Figure 7B). Similarly, compared with WT, the seed setting rate of *OsNAR2.1* RNAi lines decreased by 14.3% and that of *OsNAR2.1* over-expression lines increased by 5.6% in fully irrigated field. Further, that of *OsNAR2.1* RNAi lines was decreased by 45.0% and that of *OsNAR2.1* over-expression lines was increased by 16.3% under limited water supply conditions (Figure 7C).

The grain yield of rice is the result of various factors (Kim et al., 2009; Guan et al., 2010). In our experiments, changes in photosynthetic rate, water use efficiency, tiller number, grain number per panicle and seed setting rate led to changes in rice yield. Finally, compared with WT, the grain yield of *OsNAR2.1* RNAi lines decreased by 18.2% and that of *OsNAR2.1* over-expression lines increased by 9.1% under full watering conditions (Figure 7D). Further, *OsNAR2.1* RNAi lines decreased by 65.2% and that of *OsNAR2.1* over-expression lines increased by 26.6% under limited water supply (Figure 7D). In conclusion, overexpression of *OsNAR2.1* could improve the drought resistance without causing adverse growth phenotypes in rice.

## Conclusion

We confirmed that *OsNAR2.1* is involved in drought stress tolerance through regulating drought stress sensitivity and stress-related gene expression in rice, overexpression of *OsNRT2.1* or *OsNRT2.3a* alone could not improve rice tolerance to drought. The reason may be that overexpression of *OsNAR2.1* can significantly increase nitrogen uptake in rice, and does not reduce the ratio of ^15^NH_4_^+^ to ^15^NO_3_^−^ influx, while overexpression of *OsNRT2.1* or *OsNRT2.3a* alone cannot improve nitrogen uptake or reduce the ratio of ^15^NH_4_^+^ to ^15^NO_3_^−^ influx in rice. After variation in water supply, we found that even in limited water availability, *OsNAR2.1* over-expression significantly increased grain yield as compared to WT and led to minimal growth defects in drought stress. These results indicate that over-expression of *OsNAR2.1* is a promising strategy to improve abiotic tolerance, especially to drought in rice.

## Author Contributions

JC, GX, and XgF conceived and designed the experiments. JC, TQ, ZH, XuF, and LZ performed the experiments. JC, MI, and XY analyzed the data. JC, MI, and XgF wrote and revised the manuscript. All authors read and approved the final manuscript.

## Conflict of Interest Statement

The authors declare that the research was conducted in the absence of any commercial or financial relationships that could be construed as a potential conflict of interest.
