# Supplementary material for: OsNAR2.1 Positively Regulates Drought Tolerance and Grain Yield Under Drought Stress Conditions in Rice
Source: Front Plant Sci. 2019 Feb 21;10:197. doi: 10.3389/fpls.2019.00197 (PMC6393350; doi:10.3389/fpls.2019.00197)
Supplement: Supplementary file 1 [file Presentation_1.PPT]

## Slide 1
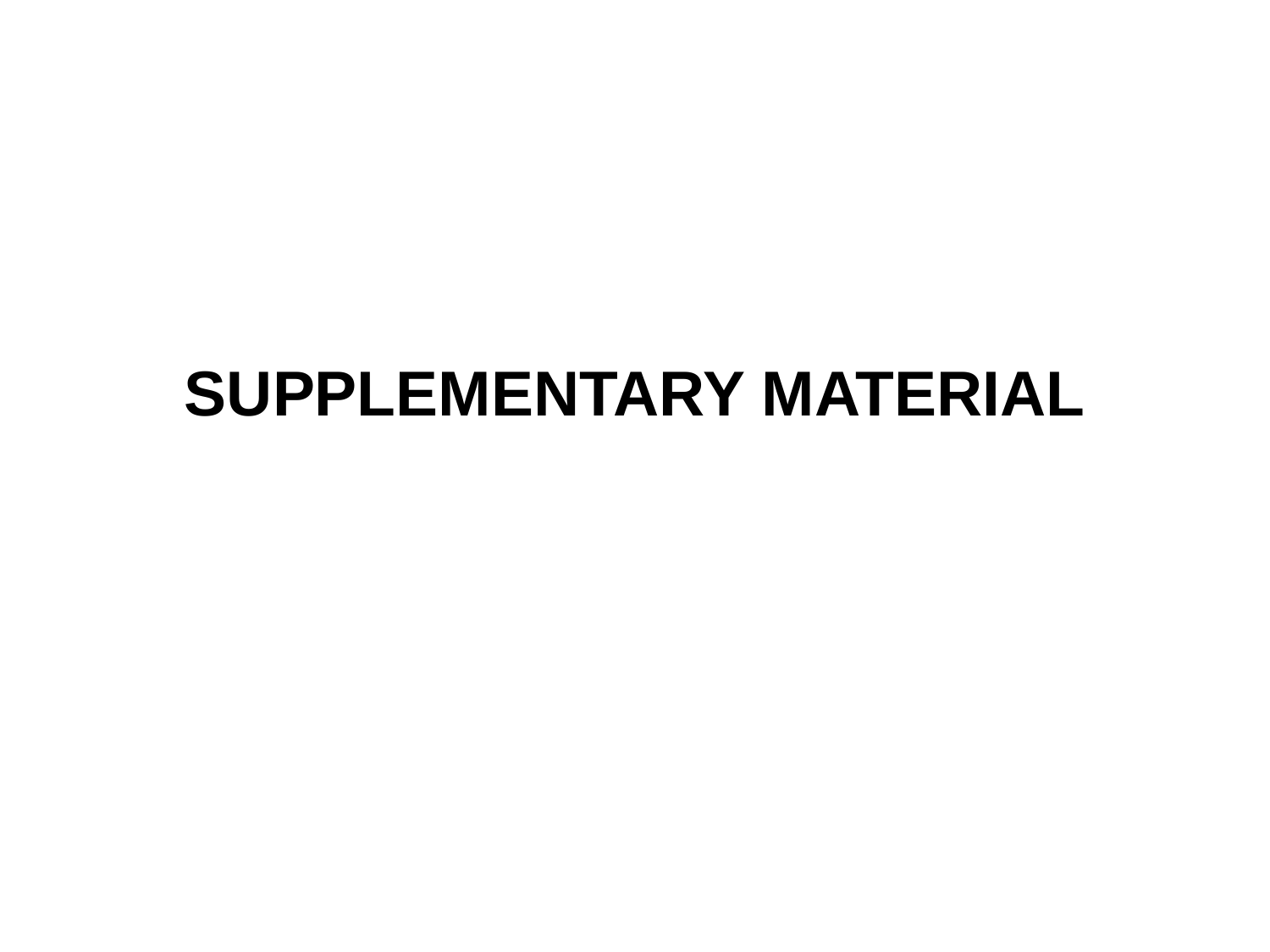

SUPPLEMENTARY MATERIAL

## Slide 2
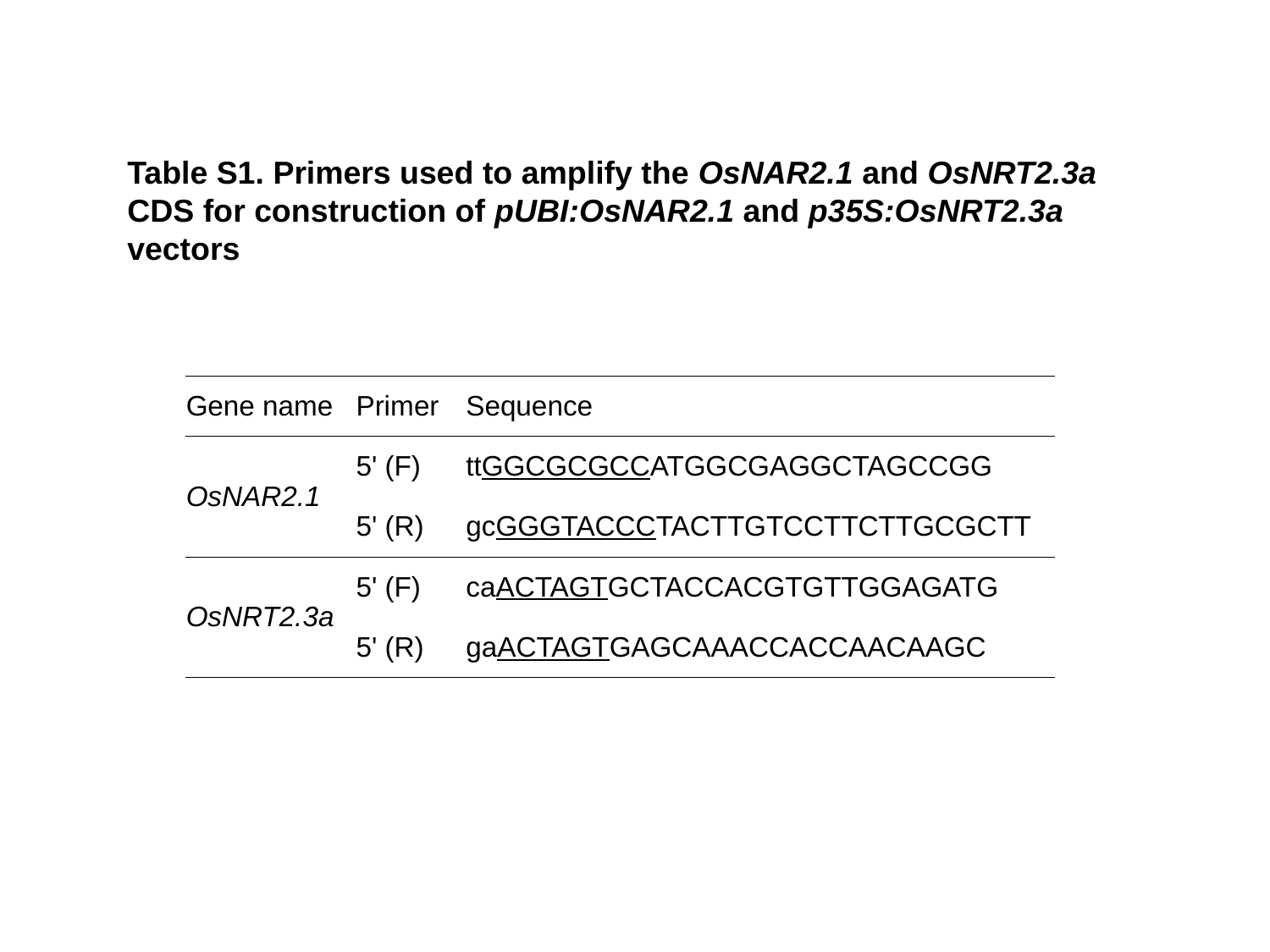

Table S1. Primers used to amplify the OsNAR2.1 and OsNRT2.3a CDS for construction of pUBI:OsNAR2.1 and p35S:OsNRT2.3a vectors
| Gene name | Primer | Sequence |
| --- | --- | --- |
| OsNAR2.1 | 5' (F) | ttGGCGCGCCATGGCGAGGCTAGCCGG |
| | 5' (R) | gcGGGTACCCTACTTGTCCTTCTTGCGCTT |
| OsNRT2.3a | 5' (F) | caACTAGTGCTACCACGTGTTGGAGATG |
| | 5' (R) | gaACTAGTGAGCAAACCACCAACAAGC |

## Slide 3
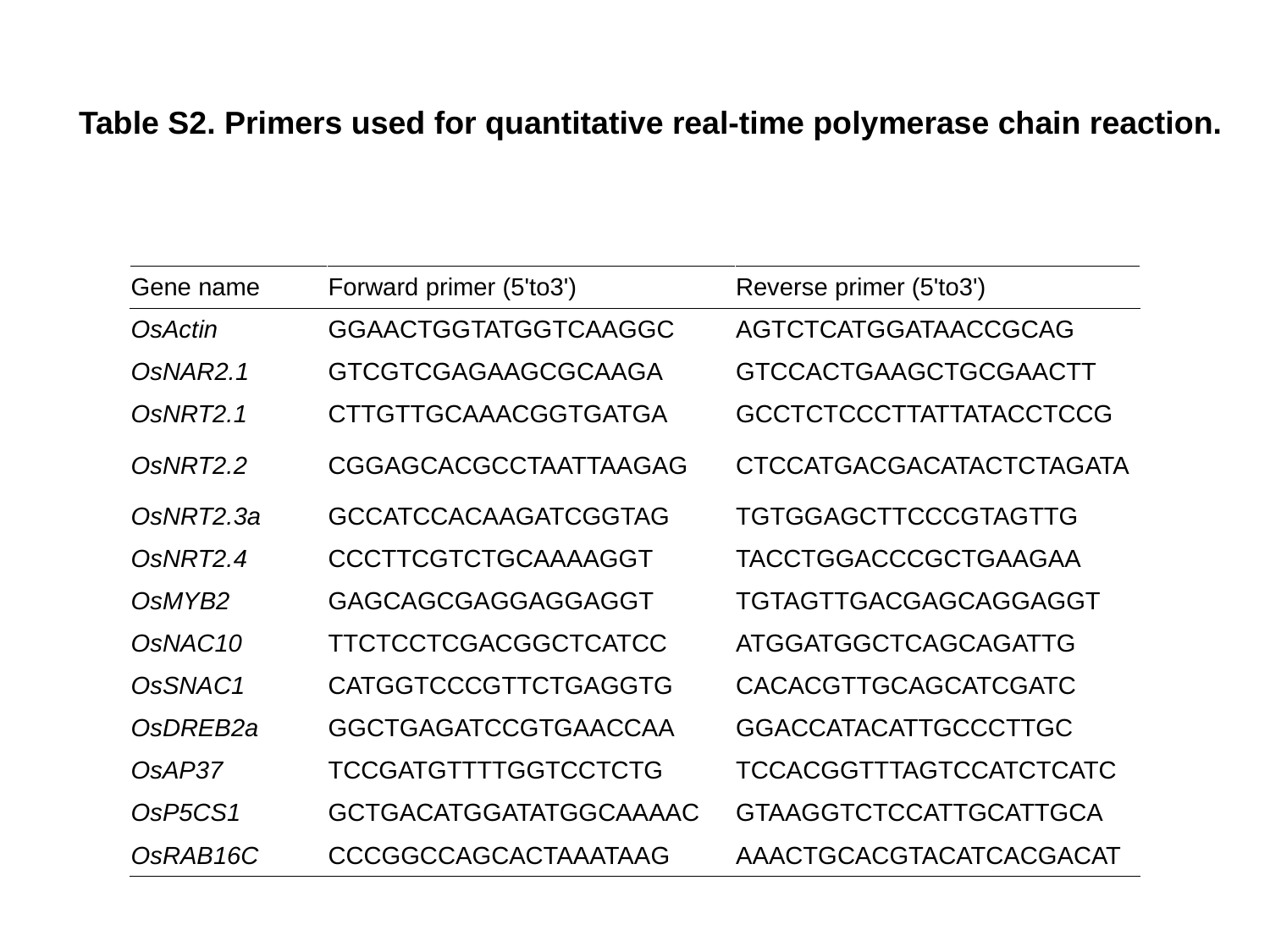

Table S2. Primers used for quantitative real-time polymerase chain reaction.
| Gene name | Forward primer (5'to3') | Reverse primer (5'to3') |
| --- | --- | --- |
| OsActin | GGAACTGGTATGGTCAAGGC | AGTCTCATGGATAACCGCAG |
| OsNAR2.1 | GTCGTCGAGAAGCGCAAGA | GTCCACTGAAGCTGCGAACTT |
| OsNRT2.1 | CTTGTTGCAAACGGTGATGA | GCCTCTCCCTTATTATACCTCCG |
| OsNRT2.2 | CGGAGCACGCCTAATTAAGAG | CTCCATGACGACATACTCTAGATA |
| OsNRT2.3a | GCCATCCACAAGATCGGTAG | TGTGGAGCTTCCCGTAGTTG |
| OsNRT2.4 | CCCTTCGTCTGCAAAAGGT | TACCTGGACCCGCTGAAGAA |
| OsMYB2 | GAGCAGCGAGGAGGAGGT | TGTAGTTGACGAGCAGGAGGT |
| OsNAC10 | TTCTCCTCGACGGCTCATCC | ATGGATGGCTCAGCAGATTG |
| OsSNAC1 | CATGGTCCCGTTCTGAGGTG | CACACGTTGCAGCATCGATC |
| OsDREB2a | GGCTGAGATCCGTGAACCAA | GGACCATACATTGCCCTTGC |
| OsAP37 | TCCGATGTTTTGGTCCTCTG | TCCACGGTTTAGTCCATCTCATC |
| OsP5CS1 | GCTGACATGGATATGGCAAAAC | GTAAGGTCTCCATTGCATTGCA |
| OsRAB16C | CCCGGCCAGCACTAAATAAG | AAACTGCACGTACATCACGACAT |

## Slide 4
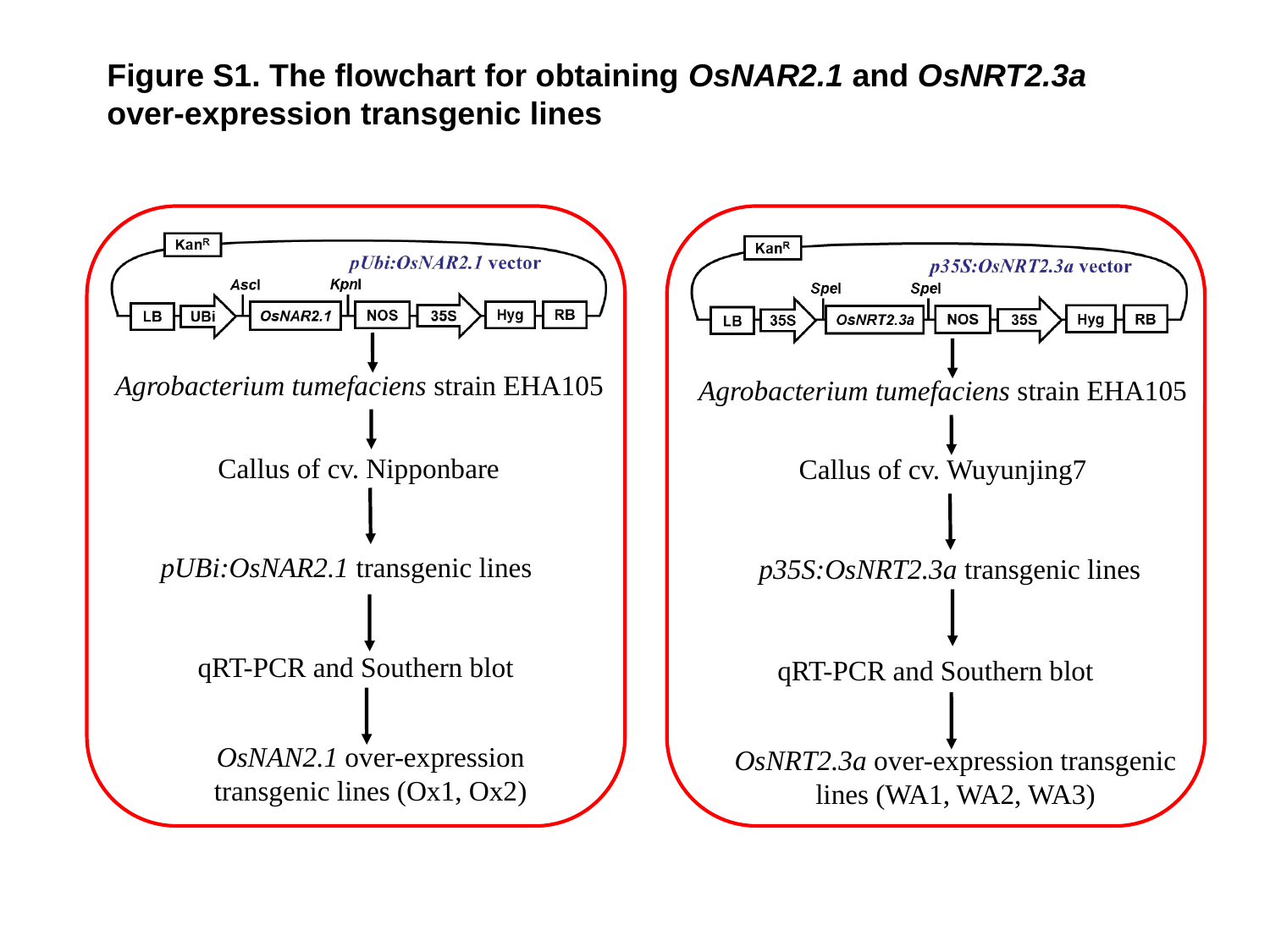

Figure S1. The flowchart for obtaining OsNAR2.1 and OsNRT2.3a over-expression transgenic lines
Agrobacterium tumefaciens strain EHA105
Agrobacterium tumefaciens strain EHA105
Callus of cv. Nipponbare
Callus of cv. Wuyunjing7
pUBi:OsNAR2.1 transgenic lines
p35S:OsNRT2.3a transgenic lines
qRT-PCR and Southern blot
qRT-PCR and Southern blot
OsNAN2.1 over-expression transgenic lines (Ox1, Ox2)
OsNRT2.3a over-expression transgenic lines (WA1, WA2, WA3)

## Slide 5
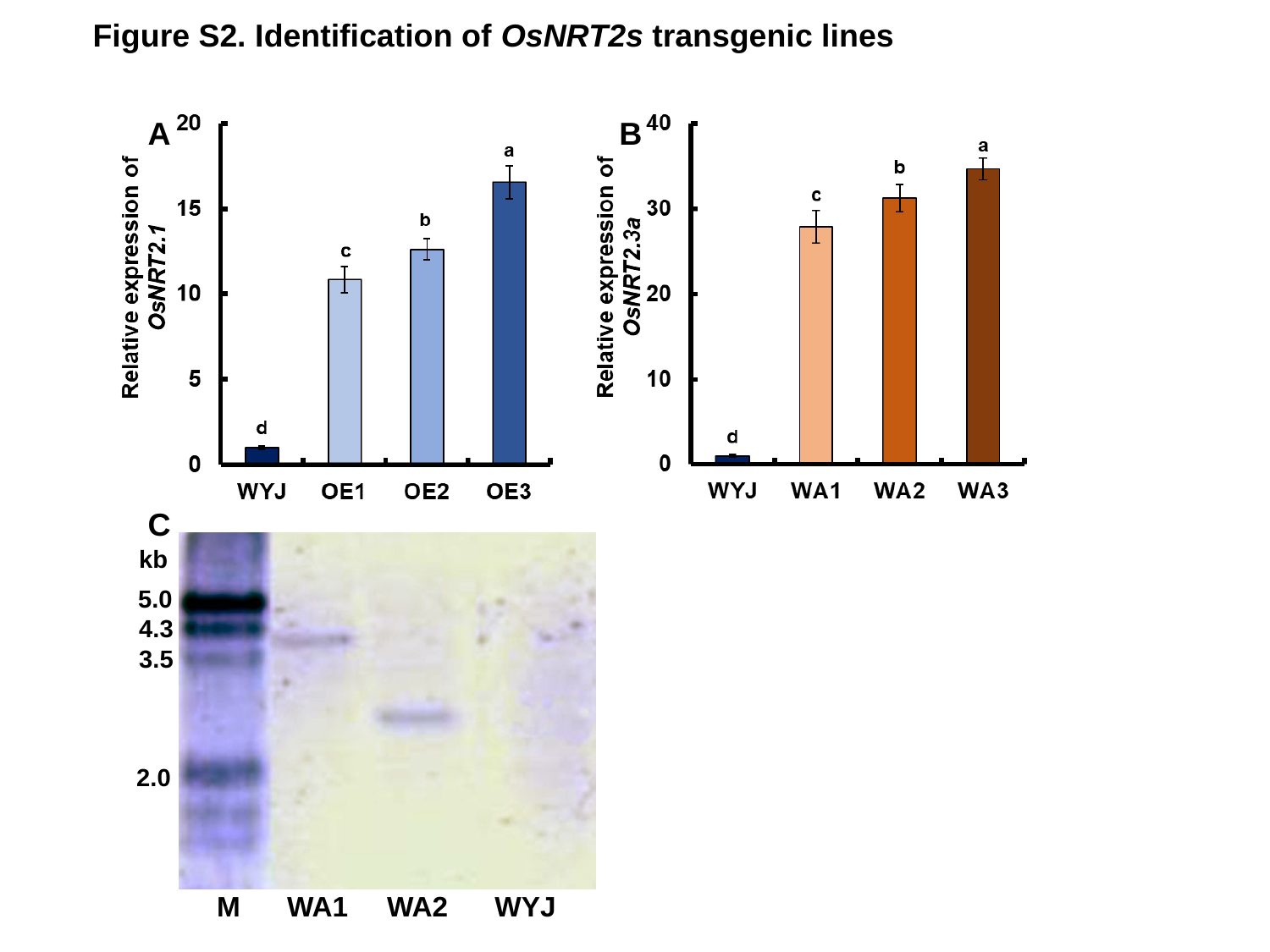

Figure S2. Identification of OsNRT2s transgenic lines
A
B
C
 M WA1 WA2 WYJ
kb
5.0
4.3
3.5
2.0

## Slide 6
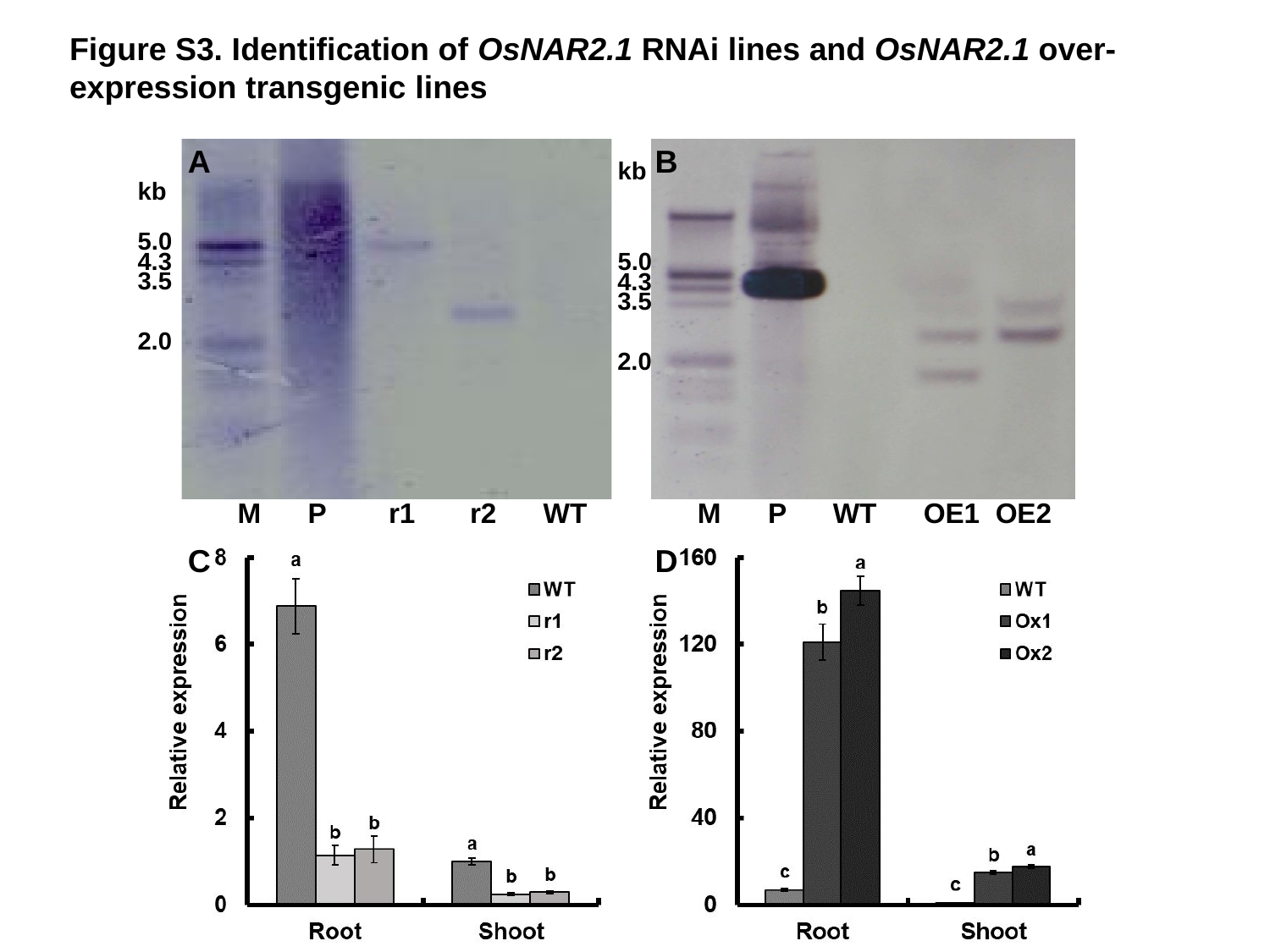

Figure S3. Identification of OsNAR2.1 RNAi lines and OsNAR2.1 over-expression transgenic lines
A
B
kb
5.0
4.3
3.5
2.0
kb
5.0
4.3
3.5
2.0
M P r1 r2 WT
M P WT OE1 OE2
C
D

## Slide 7
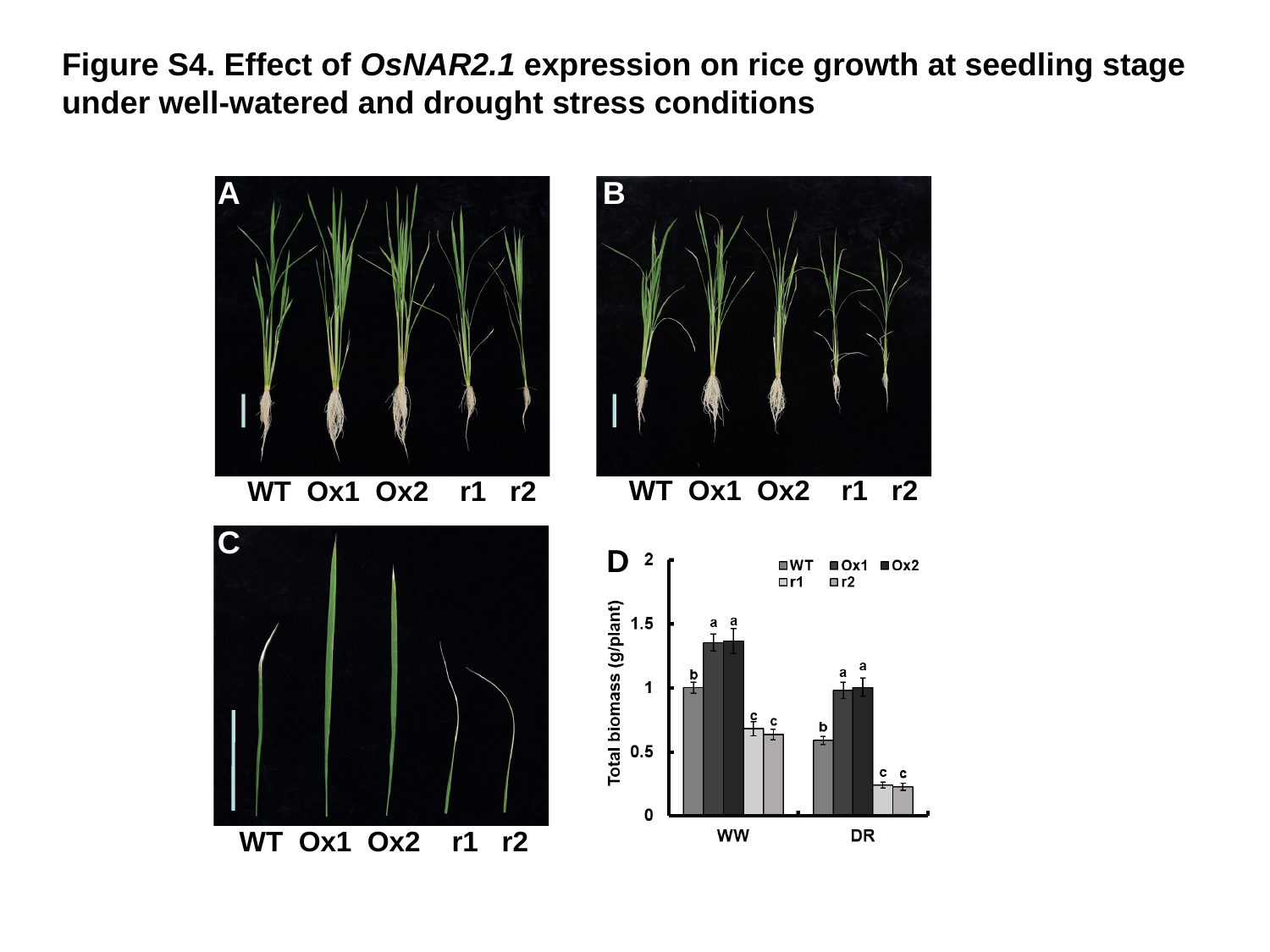

Figure S4. Effect of OsNAR2.1 expression on rice growth at seedling stage under well-watered and drought stress conditions
A
B
WT Ox1 Ox2 r1 r2
WT Ox1 Ox2 r1 r2
C
D
WT Ox1 Ox2 r1 r2

## Slide 8
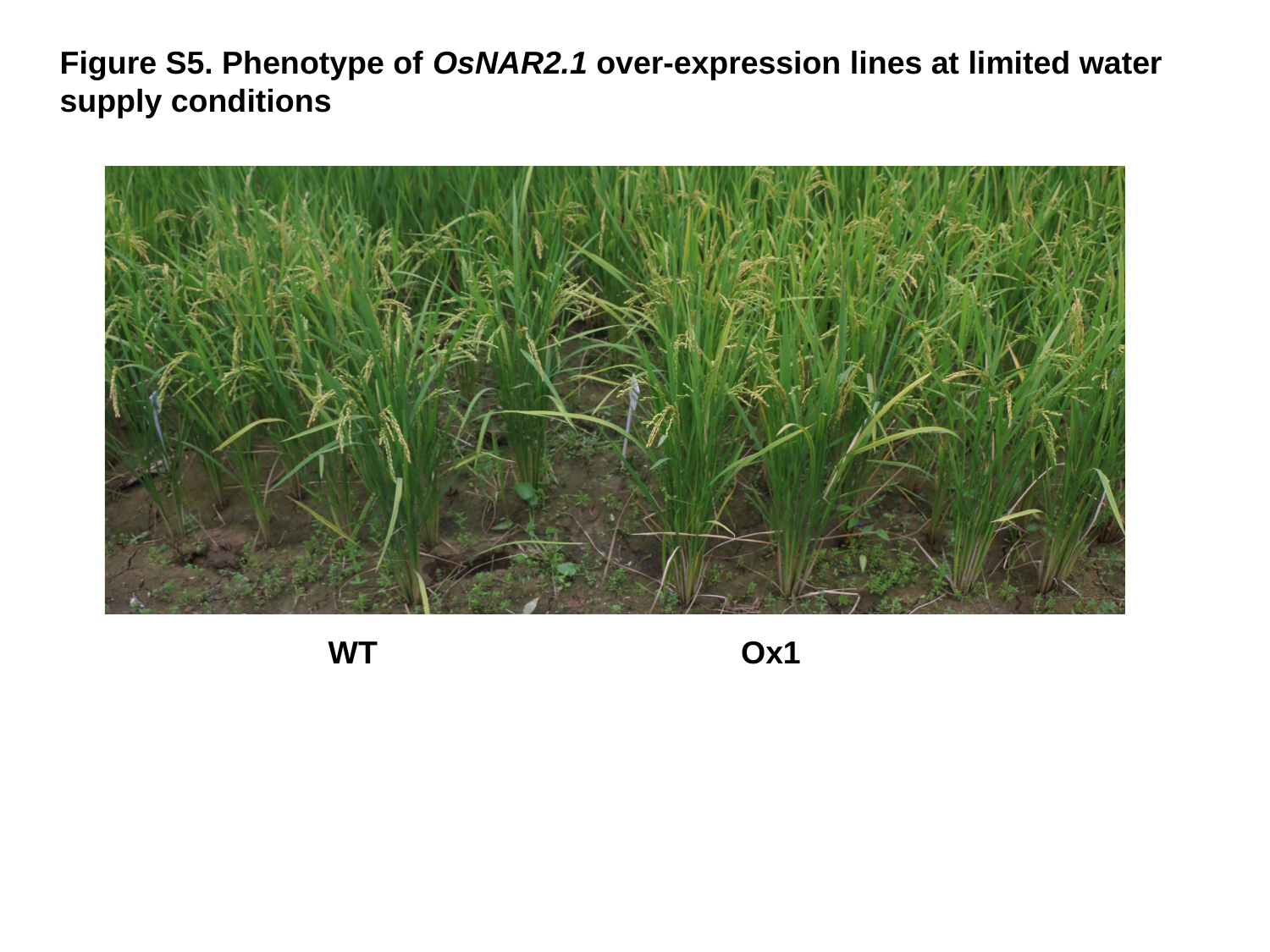

Figure S5. Phenotype of OsNAR2.1 over-expression lines at limited water supply conditions
WT Ox1

## Slide 9
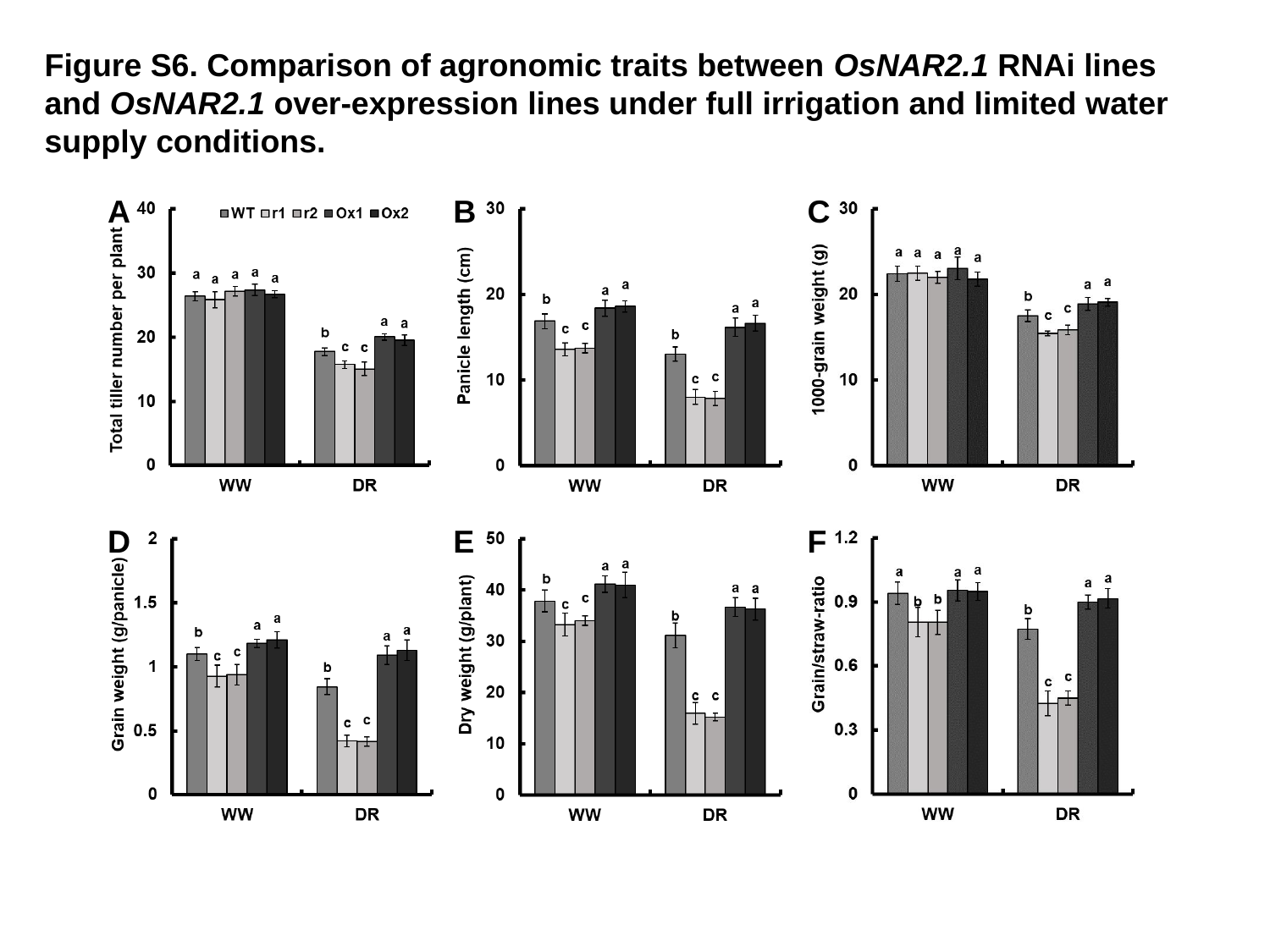

Figure S6. Comparison of agronomic traits between OsNAR2.1 RNAi lines and OsNAR2.1 over-expression lines under full irrigation and limited water supply conditions.
A
B
C
D
E
F

## Slide 10
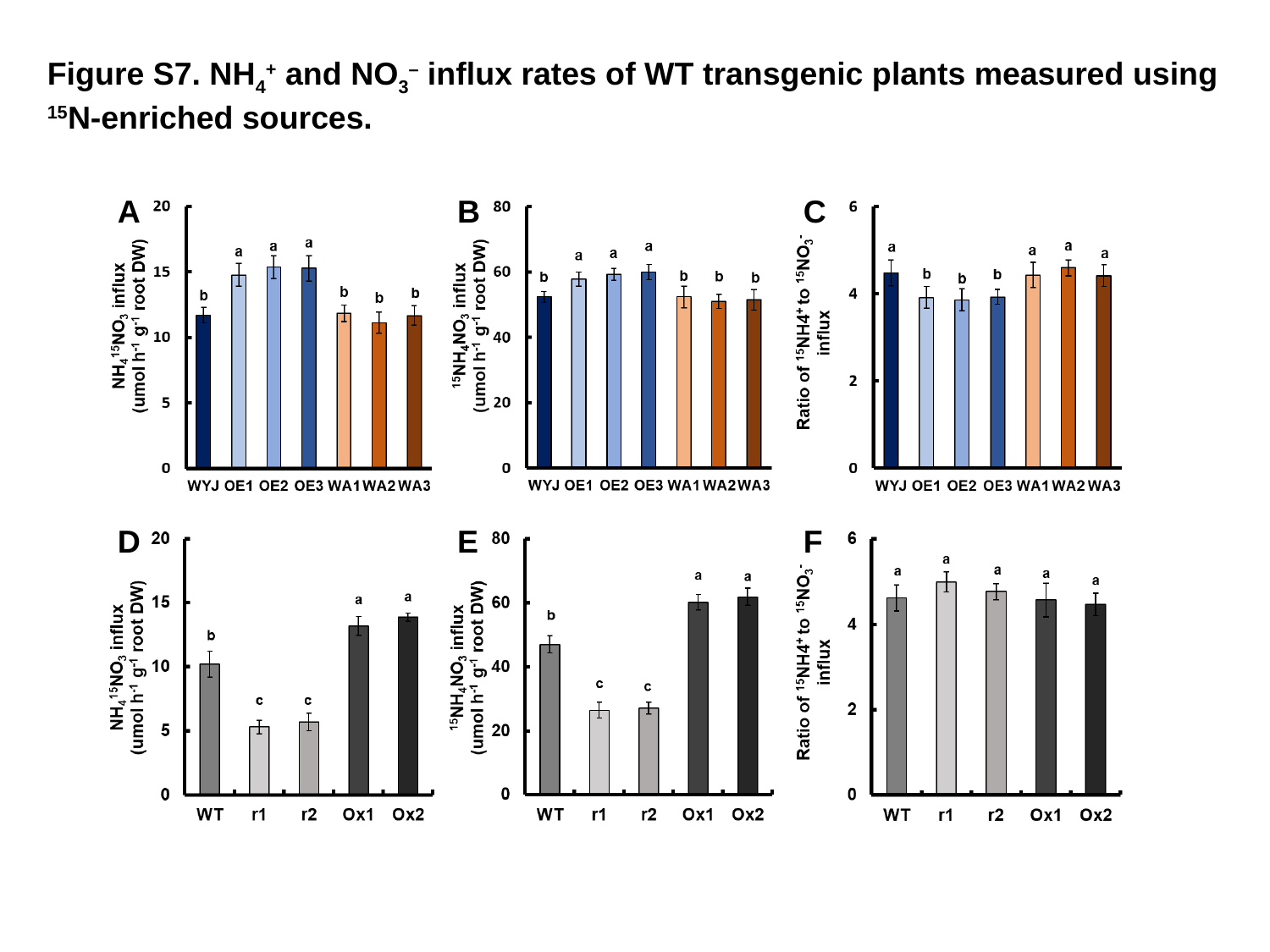

Figure S7. NH4+ and NO3– influx rates of WT transgenic plants measured using 15N-enriched sources.
A
B
C
D
E
F

## Slide 11
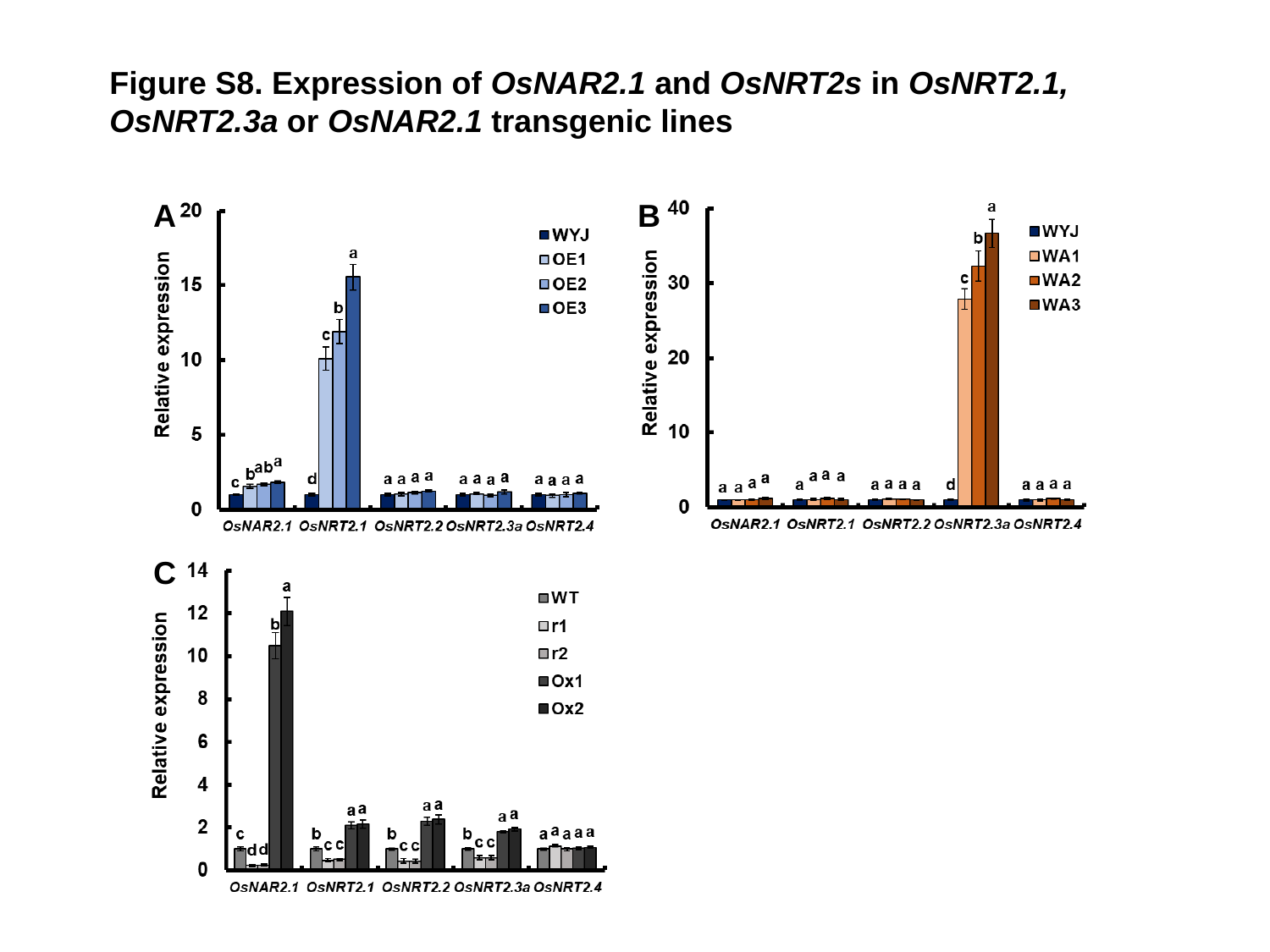

Figure S8. Expression of OsNAR2.1 and OsNRT2s in OsNRT2.1, OsNRT2.3a or OsNAR2.1 transgenic lines
A
B
C
